# 1,5-Bis(4-chloro­phen­yl)-3-[4-(dimethyl­amino)phen­yl]pentane-1,5-dione

**DOI:** 10.1107/S1600536809003894

**Published:** 2009-02-11

**Authors:** Xinyou Lei, Xiaohua Bai

**Affiliations:** a College of Life Sciences and Chemistry, Tianshui Normal University, Tianshui 741000, People’s Republic of China

## Abstract

In the title mol­ecule, C_25_H_23_Cl_2_NO_2_, the central benzene ring forms dihedral angles of 81.88 (7) and 89.22 (7)° with the two 4-chloro­phenyl fragments. The crystal packing exhibits weak inter­molecular C—H⋯O hydrogen bonds and π–π inter­actions [centroid–centroid distance 3.724 (3) Å].

## Related literature

For the crystal structures of related compounds, see: Das *et al.* (1994 ▶); Huang *et al.* (2006 ▶).
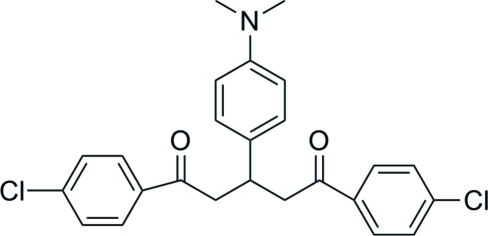


## Experimental

### 

#### Crystal data

C_25_H_23_Cl_2_NO_2_
*M* *_r_* = 440.34
Triclinic, 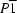

*a* = 6.1059 (11) Å
*b* = 12.5660 (16) Å
*c* = 14.731 (2) Å
α = 75.516 (1)°
β = 85.953 (2)°
γ = 87.215 (2)°
*V* = 1091.1 (3) Å^3^
*Z* = 2
Mo *K*α radiation
μ = 0.32 mm^−1^
*T* = 298 (2) K
0.49 × 0.44 × 0.41 mm

#### Data collection

Bruker SMART APEX CCD area-detector diffractometer
Absorption correction: multi-scan (*SADABS*; Sheldrick, 1996 ▶) *T*_min_ = 0.859, *T*_max_ = 0.880
5699 measured reflections
3783 independent reflections
1846 reflections with *I* > 2σ(*I*)
*R*_int_ = 0.024

#### Refinement

*R*[*F* ^2^ > 2σ(*F* ^2^)] = 0.051
*wR*(*F* ^2^) = 0.141
*S* = 0.99
3783 reflections
273 parameters
H-atom parameters constrained
Δρ_max_ = 0.18 e Å^−3^
Δρ_min_ = −0.28 e Å^−3^

### 

Data collection: *SMART* (Siemens, 1996 ▶); cell refinement: *SAINT* (Siemens, 1996 ▶); data reduction: *SAINT*; program(s) used to solve structure: *SHELXS97* (Sheldrick, 2008 ▶); program(s) used to refine structure: *SHELXL97* (Sheldrick, 2008 ▶); molecular graphics: *SHELXTL* (Sheldrick, 2008 ▶); software used to prepare material for publication: *SHELXTL*.

## Supplementary Material

Crystal structure: contains datablocks I, global. DOI: 10.1107/S1600536809003894/cv2508sup1.cif

Structure factors: contains datablocks I. DOI: 10.1107/S1600536809003894/cv2508Isup2.hkl

Additional supplementary materials:  crystallographic information; 3D view; checkCIF report

## Figures and Tables

**Table 1:** Hydrogen-bond geometry (Å, °)

*D*—H⋯*A*	*D*—H	H⋯*A*	*D*⋯*A*	*D*—H⋯*A*
C7—H7⋯O1^i^	0.93	2.58	3.176 (4)	122

Symmetry code: (i) 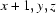.

**Fig. 1.**
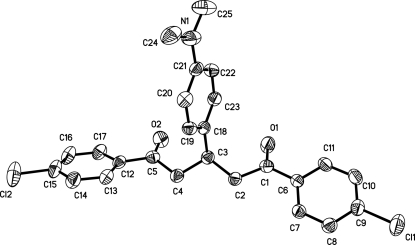
The molecular structure of (I) showing the atomic numbering scheme and 30% probability displacement ellipsoids. H atoms have been omitted for clarity.

## supplementary crystallographic information

### Comment

In this paper, we present the crystal structure of the title compound, (I), obtained with the  solvent-free protocol for the synthesis of 1,5-diketones starting from the fragrant aldehydes and fragrant ketones in the presence of NaOH under solvent-free conditions.

In (I) (Fig. 1), the bond lengths and angles are normal and correspond to those observed in 1,3,5-triphenyl-pentane-1,5-diketone (Das *et al.*, 1994), 1,5-Diphenyl-3-(2-pyridyl)pentane-1,5-dione (Huang *et al.*,  2006). The dihedral angles between the central benzene ring and the benzene rings C12/C13/C14/C15/C16/C17 and C6/C7/C8/C9/C10/C11 are 89.22 (7) ° and 81.88 (7) °, respectively. The crystal packing exhibits weak intermolecular π–π interactions (Table 1) and C—H···O hydrogen bonds (Table 2).

### Experimental

4-(Dimethylamino)benzaldehyde (0.5 mmol) and 4-chloroacetophenone (1.0 mmol), NaOH (1.0 mmol) were mixed in 50 ml flask under sovlent-free condtions. After stirring for 5 min at 293 K, the resulting mixture was washed with water for several times for removing NaOH, and recrystalized from ethanol, and afforded the title compound as a crystalline solid. Elemental analysis: calculated for C_25_H_23_Cl_2_NO_2_: C 68.19, H 5.26, N 3.18%; found: C 68.23, H 5.35, N 3.15%.

### Refinement

All H atoms were positioned geometrically and refined using a riding model with C—H = 0.93–0.98 Å and *U*_iso_(H) = 1.2-1.5*U*_eq_(C).

### Crystal data

**Table:**

C_25_H_23_Cl_2_NO_2_	*Z* = 2
*M**_r_* = 440.34	*F*(000) = 460
Triclinic, *P*1	*D*_x_ = 1.340 Mg m^−^^3^
*a* = 6.1059 (11) Å	Mo *K*α radiation, λ = 0.71073 Å
*b* = 12.5660 (16) Å	Cell parameters from 1234 reflections
*c* = 14.731 (2) Å	θ = 2.5–22.3°
α = 75.516 (1)°	µ = 0.32 mm^−^^1^
β = 85.953 (2)°	*T* = 298 K
γ = 87.215 (2)°	Block, colourless
*V* = 1091.1 (3) Å^3^	0.49 × 0.44 × 0.41 mm

### Data collection

**Table:**

Bruker SMART APEX CCD area-detector diffractometer	3783 independent reflections
Radiation source: fine-focus sealed tube	1846 reflections with *I* > 2σ(*I*)
graphite	*R*_int_ = 0.024
φ and ω scans	θ_max_ = 25.0°, θ_min_ = 1.4°
Absorption correction: multi-scan (*SADABS*; Sheldrick, 1996)	*h* = −7→7
*T*_min_ = 0.859, *T*_max_ = 0.880	*k* = −8→14
5699 measured reflections	*l* = −17→17

### Refinement

**Table:**

Refinement on *F*^2^	Primary atom site location: structure-invariant direct methods
Least-squares matrix: full	Secondary atom site location: difference Fourier map
*R*[*F*^2^ > 2σ(*F*^2^)] = 0.051	Hydrogen site location: inferred from neighbouring sites
*wR*(*F*^2^) = 0.141	H-atom parameters constrained
*S* = 0.99	*w* = 1/[σ^2^(*F*_o_^2^) + (0.0595*P*)^2^] where *P* = (*F*_o_^2^ + 2*F*_c_^2^)/3
3783 reflections	(Δ/σ)_max_ = 0.001
273 parameters	Δρ_max_ = 0.18 e Å^−^^3^
0 restraints	Δρ_min_ = −0.28 e Å^−^^3^

### Special details

**Table:**

Geometry. All e.s.d.'s (except the e.s.d. in the dihedral angle between two l.s. planes) are estimated using the full covariance matrix. The cell e.s.d.'s are taken into account individually in the estimation of e.s.d.'s in distances, angles and torsion angles; correlations between e.s.d.'s in cell parameters are only used when they are defined by crystal symmetry. An approximate (isotropic) treatment of cell e.s.d.'s is used for estimating e.s.d.'s involving l.s. planes.
Refinement. Refinement of *F*^2^ against ALL reflections. The weighted *R*-factor *wR* and goodness of fit *S* are based on *F*^2^, conventional *R*-factors *R* are based on *F*, with *F* set to zero for negative *F*^2^. The threshold expression of *F*^2^ > σ(*F*^2^) is used only for calculating *R*-factors(gt) *etc*. and is not relevant to the choice of reflections for refinement. *R*-factors based on *F*^2^ are statistically about twice as large as those based on *F*, and *R*- factors based on ALL data will be even larger.

### Fractional atomic coordinates and isotropic or equivalent isotropic displacement parameters (Å^2^)

**Table:**

	*x*	*y*	*z*	*U*_iso_*/*U*_eq_	
Cl1	1.1281 (2)	0.35929 (8)	0.70584 (7)	0.1057 (4)	
Cl2	0.8066 (2)	1.57712 (9)	0.06677 (8)	0.1334 (5)	
N1	0.5447 (5)	0.8356 (3)	0.0253 (2)	0.0836 (9)	
O1	0.4561 (4)	0.75133 (19)	0.48581 (16)	0.0838 (8)	
O2	0.3440 (4)	1.12960 (19)	0.32504 (17)	0.0811 (7)	
C1	0.6537 (6)	0.7566 (3)	0.4892 (2)	0.0550 (8)	
C2	0.7662 (5)	0.8611 (2)	0.4432 (2)	0.0560 (8)	
H2A	0.8980	0.8441	0.4080	0.067*	
H2B	0.8109	0.8943	0.4914	0.067*	
C3	0.6236 (5)	0.9442 (2)	0.3771 (2)	0.0551 (8)	
H3	0.4755	0.9419	0.4076	0.066*	
C4	0.7007 (5)	1.0611 (2)	0.3626 (2)	0.0587 (9)	
H4A	0.7239	1.0744	0.4232	0.070*	
H4B	0.8403	1.0685	0.3262	0.070*	
C5	0.5388 (6)	1.1462 (3)	0.3128 (2)	0.0556 (8)	
C6	0.7791 (5)	0.6593 (2)	0.54189 (19)	0.0486 (8)	
C7	0.9857 (5)	0.6658 (3)	0.5707 (2)	0.0573 (8)	
H7	1.0549	0.7328	0.5548	0.069*	
C8	1.0912 (6)	0.5741 (3)	0.6229 (2)	0.0667 (9)	
H8	1.2297	0.5793	0.6437	0.080*	
C9	0.9908 (7)	0.4750 (3)	0.6441 (2)	0.0650 (9)	
C10	0.7867 (7)	0.4664 (3)	0.6155 (2)	0.0744 (10)	
H10	0.7202	0.3987	0.6294	0.089*	
C11	0.6806 (6)	0.5591 (3)	0.5657 (2)	0.0635 (9)	
H11	0.5392	0.5541	0.5476	0.076*	
C12	0.6156 (6)	1.2513 (3)	0.2519 (2)	0.0546 (8)	
C13	0.8291 (6)	1.2674 (3)	0.2151 (2)	0.0632 (9)	
H13	0.9347	1.2109	0.2298	0.076*	
C14	0.8859 (6)	1.3673 (3)	0.1565 (2)	0.0779 (11)	
H14	1.0288	1.3779	0.1304	0.093*	
C15	0.7308 (8)	1.4511 (3)	0.1368 (2)	0.0804 (11)	
C16	0.5199 (8)	1.4378 (3)	0.1735 (2)	0.0854 (12)	
H16	0.4164	1.4955	0.1604	0.102*	
C17	0.4640 (6)	1.3385 (3)	0.2298 (2)	0.0707 (10)	
H17	0.3198	1.3286	0.2544	0.085*	
C18	0.6048 (5)	0.9143 (2)	0.2842 (2)	0.0490 (8)	
C19	0.7708 (5)	0.9307 (2)	0.2150 (2)	0.0572 (8)	
H19	0.9003	0.9603	0.2258	0.069*	
C20	0.7540 (6)	0.9053 (3)	0.1304 (2)	0.0632 (9)	
H20	0.8705	0.9189	0.0853	0.076*	
C21	0.5653 (6)	0.8595 (3)	0.1113 (2)	0.0596 (9)	
C22	0.3988 (6)	0.8402 (3)	0.1817 (2)	0.0663 (9)	
H22	0.2711	0.8079	0.1723	0.080*	
C23	0.4195 (5)	0.8681 (2)	0.2655 (2)	0.0597 (9)	
H23	0.3035	0.8551	0.3109	0.072*	
C24	0.7091 (7)	0.8654 (3)	−0.0499 (3)	0.0939 (13)	
H24A	0.7429	0.9413	−0.0588	0.141*	
H24B	0.6554	0.8546	−0.1066	0.141*	
H24C	0.8393	0.8202	−0.0348	0.141*	
C25	0.3520 (7)	0.7852 (4)	0.0080 (3)	0.1137 (16)	
H25A	0.3125	0.7267	0.0617	0.171*	
H25B	0.3806	0.7559	−0.0461	0.171*	
H25C	0.2334	0.8390	−0.0031	0.171*	

### Atomic displacement parameters (Å^2^)

**Table:**

	*U*^11^	*U*^22^	*U*^33^	*U*^12^	*U*^13^	*U*^23^
Cl1	0.1403 (11)	0.0722 (7)	0.0954 (8)	0.0259 (6)	−0.0218 (7)	−0.0052 (6)
Cl2	0.1990 (14)	0.0788 (8)	0.0976 (9)	0.0017 (8)	0.0142 (9)	0.0169 (6)
N1	0.092 (3)	0.096 (2)	0.072 (2)	0.0026 (19)	−0.0208 (19)	−0.0359 (19)
O1	0.0554 (16)	0.0829 (18)	0.0964 (19)	−0.0125 (13)	−0.0049 (14)	0.0110 (14)
O2	0.0617 (17)	0.0758 (18)	0.103 (2)	0.0036 (14)	−0.0004 (14)	−0.0203 (14)
C1	0.050 (2)	0.068 (2)	0.0468 (19)	−0.0123 (18)	0.0025 (16)	−0.0132 (17)
C2	0.059 (2)	0.060 (2)	0.0468 (18)	−0.0079 (17)	−0.0023 (16)	−0.0077 (16)
C3	0.054 (2)	0.057 (2)	0.052 (2)	−0.0022 (16)	−0.0017 (16)	−0.0100 (16)
C4	0.069 (2)	0.057 (2)	0.0517 (19)	0.0013 (18)	−0.0075 (17)	−0.0152 (16)
C5	0.059 (2)	0.060 (2)	0.053 (2)	0.0031 (19)	−0.0031 (18)	−0.0243 (18)
C6	0.051 (2)	0.053 (2)	0.0420 (18)	−0.0077 (17)	0.0049 (15)	−0.0128 (15)
C7	0.053 (2)	0.057 (2)	0.061 (2)	−0.0062 (17)	−0.0018 (17)	−0.0117 (17)
C8	0.063 (2)	0.069 (3)	0.067 (2)	−0.001 (2)	−0.0065 (18)	−0.015 (2)
C9	0.088 (3)	0.054 (2)	0.052 (2)	0.007 (2)	−0.0023 (19)	−0.0143 (18)
C10	0.095 (3)	0.054 (2)	0.076 (3)	−0.017 (2)	−0.004 (2)	−0.017 (2)
C11	0.066 (2)	0.058 (2)	0.069 (2)	−0.0129 (19)	−0.0041 (19)	−0.0183 (19)
C12	0.067 (2)	0.055 (2)	0.0439 (19)	0.0068 (18)	−0.0083 (17)	−0.0152 (16)
C13	0.070 (3)	0.063 (2)	0.053 (2)	0.0080 (19)	−0.0059 (18)	−0.0078 (18)
C14	0.083 (3)	0.083 (3)	0.061 (2)	−0.002 (2)	−0.002 (2)	−0.005 (2)
C15	0.112 (4)	0.064 (3)	0.054 (2)	0.009 (2)	−0.002 (2)	0.0012 (19)
C16	0.110 (4)	0.074 (3)	0.061 (2)	0.027 (2)	−0.007 (2)	−0.002 (2)
C17	0.079 (3)	0.075 (3)	0.054 (2)	0.014 (2)	−0.0021 (19)	−0.012 (2)
C18	0.048 (2)	0.0485 (19)	0.0488 (19)	0.0037 (15)	−0.0034 (16)	−0.0098 (15)
C19	0.052 (2)	0.061 (2)	0.061 (2)	−0.0015 (16)	−0.0095 (17)	−0.0180 (17)
C20	0.060 (2)	0.069 (2)	0.059 (2)	0.0039 (18)	0.0010 (17)	−0.0168 (18)
C21	0.071 (2)	0.052 (2)	0.056 (2)	0.0113 (18)	−0.0178 (19)	−0.0124 (17)
C22	0.063 (2)	0.065 (2)	0.073 (3)	−0.0064 (18)	−0.018 (2)	−0.0177 (19)
C23	0.052 (2)	0.064 (2)	0.060 (2)	−0.0028 (17)	−0.0035 (17)	−0.0077 (18)
C24	0.114 (3)	0.109 (3)	0.063 (3)	0.018 (3)	−0.012 (2)	−0.032 (2)
C25	0.107 (4)	0.145 (4)	0.115 (4)	0.007 (3)	−0.039 (3)	−0.074 (3)

### Geometric parameters (Å, °)

**Table:**

Cl1—C9	1.724 (3)	C11—H11	0.9300
Cl2—C15	1.724 (4)	C12—C13	1.380 (4)
N1—C21	1.389 (4)	C12—C17	1.389 (4)
N1—C24	1.430 (4)	C13—C14	1.378 (5)
N1—C25	1.431 (4)	C13—H13	0.9300
O1—C1	1.217 (3)	C14—C15	1.371 (5)
O2—C5	1.211 (3)	C14—H14	0.9300
C1—C6	1.484 (4)	C15—C16	1.362 (5)
C1—C2	1.495 (4)	C16—C17	1.359 (5)
C2—C3	1.523 (4)	C16—H16	0.9300
C2—H2A	0.9700	C17—H17	0.9300
C2—H2B	0.9700	C18—C19	1.371 (4)
C3—C18	1.519 (4)	C18—C23	1.372 (4)
C3—C4	1.523 (4)	C19—C20	1.373 (4)
C3—H3	0.9800	C19—H19	0.9300
C4—C5	1.505 (4)	C20—C21	1.389 (4)
C4—H4A	0.9700	C20—H20	0.9300
C4—H4B	0.9700	C21—C22	1.384 (4)
C5—C12	1.476 (4)	C22—C23	1.381 (4)
C6—C7	1.371 (4)	C22—H22	0.9300
C6—C11	1.376 (4)	C23—H23	0.9300
C7—C8	1.374 (4)	C24—H24A	0.9600
C7—H7	0.9300	C24—H24B	0.9600
C8—C9	1.370 (4)	C24—H24C	0.9600
C8—H8	0.9300	C25—H25A	0.9600
C9—C10	1.361 (4)	C25—H25B	0.9600
C10—C11	1.371 (5)	C25—H25C	0.9600
C10—H10	0.9300		
			
Cg···Cg^i^	3.724 (3)		
			
C21—N1—C24	121.3 (3)	C17—C12—C5	118.1 (3)
C21—N1—C25	120.5 (3)	C14—C13—C12	120.0 (3)
C24—N1—C25	118.1 (3)	C14—C13—H13	120.0
O1—C1—C6	119.2 (3)	C12—C13—H13	120.0
O1—C1—C2	120.1 (3)	C15—C14—C13	119.7 (4)
C6—C1—C2	120.8 (3)	C15—C14—H14	120.1
C1—C2—C3	113.6 (3)	C13—C14—H14	120.1
C1—C2—H2A	108.9	C16—C15—C14	121.4 (4)
C3—C2—H2A	108.9	C16—C15—Cl2	119.4 (3)
C1—C2—H2B	108.9	C14—C15—Cl2	119.2 (4)
C3—C2—H2B	108.9	C17—C16—C15	118.6 (4)
H2A—C2—H2B	107.7	C17—C16—H16	120.7
C18—C3—C4	111.8 (2)	C15—C16—H16	120.7
C18—C3—C2	112.2 (2)	C16—C17—C12	122.0 (4)
C4—C3—C2	111.8 (2)	C16—C17—H17	119.0
C18—C3—H3	106.9	C12—C17—H17	119.0
C4—C3—H3	106.9	C19—C18—C23	116.2 (3)
C2—C3—H3	106.9	C19—C18—C3	122.4 (3)
C5—C4—C3	112.6 (3)	C23—C18—C3	121.4 (3)
C5—C4—H4A	109.1	C18—C19—C20	122.8 (3)
C3—C4—H4A	109.1	C18—C19—H19	118.6
C5—C4—H4B	109.1	C20—C19—H19	118.6
C3—C4—H4B	109.1	C19—C20—C21	120.9 (3)
H4A—C4—H4B	107.8	C19—C20—H20	119.6
O2—C5—C12	119.8 (3)	C21—C20—H20	119.6
O2—C5—C4	119.8 (3)	C22—C21—C20	116.8 (3)
C12—C5—C4	120.4 (3)	C22—C21—N1	121.9 (3)
C7—C6—C11	118.7 (3)	C20—C21—N1	121.3 (3)
C7—C6—C1	122.8 (3)	C23—C22—C21	121.0 (3)
C11—C6—C1	118.5 (3)	C23—C22—H22	119.5
C6—C7—C8	120.5 (3)	C21—C22—H22	119.5
C6—C7—H7	119.7	C18—C23—C22	122.4 (3)
C8—C7—H7	119.7	C18—C23—H23	118.8
C9—C8—C7	119.5 (3)	C22—C23—H23	118.8
C9—C8—H8	120.2	N1—C24—H24A	109.5
C7—C8—H8	120.2	N1—C24—H24B	109.5
C10—C9—C8	120.9 (3)	H24A—C24—H24B	109.5
C10—C9—Cl1	119.7 (3)	N1—C24—H24C	109.5
C8—C9—Cl1	119.3 (3)	H24A—C24—H24C	109.5
C9—C10—C11	119.0 (3)	H24B—C24—H24C	109.5
C9—C10—H10	120.5	N1—C25—H25A	109.5
C11—C10—H10	120.5	N1—C25—H25B	109.5
C10—C11—C6	121.3 (3)	H25A—C25—H25B	109.5
C10—C11—H11	119.4	N1—C25—H25C	109.5
C6—C11—H11	119.4	H25A—C25—H25C	109.5
C13—C12—C17	118.3 (3)	H25B—C25—H25C	109.5
C13—C12—C5	123.7 (3)		

Symmetry codes: (i) −*x*+2, −*y*+1, −*z*+1.

### Hydrogen-bond geometry (Å, °)

**Table:**

*D*—H···*A*	*D*—H	H···*A*	*D*···*A*	*D*—H···*A*
C7—H7···O1^ii^	0.93	2.58	3.176 (4)	122

Symmetry codes: (ii) *x*+1, *y*, *z*.
